# Does attractiveness influence condom use intentions in women who have sex with men?

**DOI:** 10.1371/journal.pone.0217152

**Published:** 2019-05-23

**Authors:** Anastasia Eleftheriou, Seth Bullock, Cynthia A. Graham, Shayna Skakoon-Sparling, Roger Ingham

**Affiliations:** 1 Electronics and Computer Science, University of Southampton, United Kingdom; 2 Institute for Complex Systems Simulation, University of Southampton, United Kingdom; 3 Department of Computer Science, University of Bristol, United Kingdom; 4 Centre for Sexual Health Research, Psychology, University of Southampton, United Kingdom; 5 Department of Family Relations and Applied Nutrition, University of Guelph, Canada; University of Pretoria, SOUTH AFRICA

## Abstract

**Objectives:**

Attractiveness judgements have been shown to affect interpersonal relationships. The present study explored the relationships between perceived attractiveness, perceived sexual health status, condom use intentions and condom use resistance in women.

**Setting:**

The study data were collected using an online questionnaire.

**Participants:**

480 English-speaking women who have sex with men, between 18–32 years old.

**Outcome measures:**

Women were asked to rate the attractiveness of 20 men on the basis of facial photographs, to estimate the likelihood that each man had a sexually transmitted infection (STI), and to indicate their willingness to have sex with each man without a condom. Condom resistance tactics were also measured and their influence on condom use intentions was assessed.

**Results:**

The more attractive a man was judged to be, the more likely it was that participants were willing to have sex with him (*r* (478) = 0.987, *p* < .001). Further, the more attractive a man was judged to be, the less likely women were to intend to use a condom during sex (*r* = -0.552, df = 478, *p* = .007). The average perceived STI likelihood for a man had no significant association with his average perceived attractiveness or with participants’ average willingness to have sex with him. The more attractive a participant judged herself to be, the more she believed that, overall, men are likely to have a STI (*r* = 0.103, df = 478, *p* < .05).

**Conclusions:**

Women’s perceptions of men’s attractiveness influence their condom use intentions; such risk biases should be incorporated into sexual health education programmes and condom use interventions.

## Introduction

Perceptions of attractiveness, both our self-perceptions and our perceptions of others, have an impact on our interpersonal relationships. Facial attractiveness, in particular, has been the subject of extensive research, as it dramatically influences the context of social interactions [1], including decisions about sexual/romantic partner selection and sexual behaviour [2] [3] [4]. Indeed, recent work [5] demonstrated a strong correlation between the perceived facial attractiveness of women and the condom use intentions of heterosexual men. In this study, men were both more interested in having sex with the more attractive female targets, and they reported lower condom use intentions for sex with the women that they found more attractive. Further demonstrating the importance of perceived facial attractiveness, these men perceived the less attractive female targets as more likely to have a sexually transmitted infection (STI) and reported higher condom use intentions when they perceived greater STI risk. These findings fit well with other work indicating an association between facial attractiveness and perceived health [6]. The current work explored women’s perceptions of male targets’ attractiveness and risk for STI transmission. We also examined women’s condom use intentions based on their perceptions of male targets in order to determine how romantic attraction may influence women’s decision about their sexual health practices with new sexual partners.

Fishbein et al. [7] and Henderson et al. [8] explored the association between romantic attraction and health risk by asking male and female participants to rate the importance of attributes that are often used to describe romantic partners, such as ‘physical build’ or ‘emotionality’. These authors found that the more a participant was attracted to a potential partner, the less likely they were to consider this person as a health risk, despite the presentation of ‘risky’ features (e.g., being unfaithful). Relatedly, Sparling and Cramer [9] also found that participants showed greater risk-taking intentions with hypothetical partners they had rated as more appealing. In fact, although women may report being more interested in having sex with low-risk men [10], they also want to have sex with more attractive men. The perceived attractiveness of a potential partner seems to play an important role: even when they believed that attractive men were more likely to carry an STI, women still report a greater willingness to have condomless sex, despite the potential risk to their sexual health [11] [12].

However, the extant work has neglected to consider the effect of one’s self-perceived attractiveness on their judgements of risk and attraction. Considering participants’ self-rated attractiveness when analysing condom use intentions may be critical; because self-perceived attractiveness could influence sexual preferences [13], perceived STI risk [14], and also mating decisions, as individuals tend to choose partners who physically resemble themselves and/or appear to have similar facial features [15]. Eleftheriou et al. [5] found that men who judged themselves to be more attractive reported lower condom use intentions overall and estimated lower rates of condom use amongst “men like themselves”. This finding corroborates research related to the Information-Motivation-Behavioural Skills model [16], which suggests that individuals’ perceptions of condom use norms in their own social group relate to their own condom use intentions. However, it is currently unknown whether women with high self-perceived attractiveness show the same attitudes and behavioral intentions. This line of questioning has important health implications as women who have sex with men are almost exclusively receptive partners and, as such, are at greater risk for STI/HIV transmission, [17].

In the current study we investigated the associations among women’s perceptions of the facial attractiveness of male targets and their perceptions of STI risk and willingness to have condomless sex. Although previous research offers some insight into the associations between facial attractiveness, perceived risk, and condom use intentions, the findings have not been entirely consistent and have not considered the possible impact of demographic variables and sexual histories of the raters. The current study extends and adds to the literature in this area by eliciting women’s condom use intentions towards men and evaluating these, not only with respect to the perceived attractiveness of the men, but also considering the participants’ self-perceived attractiveness, sexual history (including their typical condom use behaviour), and their perceptions of their peers’ (other women’s) normative condom use intentions.

We also aimed to explore the deployment of condom use resistance tactics [18] as another factor related to sexual risk taking in our sample. Perceptions about condoms and endorsement of condom resistance tactics strongly predicts consistency of condom use [19]. Further, heterosexual women who endorse condom use resistance tactics are more likely to see themselves as at lower risk for STIs, although they also tend to simultaneously report greater lifetime incidence of infection [20]. Thus, we aimed to determine whether self-perceived attractiveness and perceived partner attractiveness would be associated with greater endorsement of condom use resistance tactics.

We aimed to address the following research questions: 1. Does the perceived attractiveness of a potential sexual partner affect sex and/or condom use intentions? 2. Does a participant’s self-perceived attractiveness affect their sexual and/or condom use intentions? 3. Do demographic or sexual experience variables predict condom use intentions?

## Methods

### Participants

Data were collected online between February and April 2017. Women in the United Kingdom were recruited via social media (Facebook, Twitter), posters at a local university and on community advertisement boards, and advertisements on the university’s online participant recruitment site. Women in Canada were recruited from an Ontario university, using an advertisement posted on the course websites for a variety of first and second year courses. Eligible participants received course credits as remuneration for their participation. Potential participants were informed that data would be collected using questionnaires in order to investigate the influence of attractiveness on sexual attitudes and intentions. Eligible participants had to be between 18 and 69 years of age, English speaking, and identify as women who have sex with men. Five hundred and seventy-four women attempted the questionnaire, but 85 did not complete the full study and 9 reported being attracted to women: the final analytical sample was 480 participants.

### Measures

All data were collected using an online questionnaire in iSurvey, a University of Southampton secure online survey platform. The final questionnaire comprised four sections: 1. Participants’ demographic information and judgement of their own attractiveness, 2. Information regarding the participant’s own sexual experience and safer sex practices, 3. Five judgements of each of twenty men using a single full frontal facial photograph. The order of the 100 test items in section 3 was fully randomised for each participant. 4. The Condom Use Resistance Scale.

In the remainder of the paper, we use a series of single-letter labels to identify key variables associated with the six categories of questionnaire items introduced in parentheses on their first mention below.

#### Demographics reporting and own attractiveness ratings

Participants were asked to report their age, nationality, and occupation, and to rate their self-perceived attractiveness (*O*) on a scale from 0 to 100, where 0 indicated ‘very unattractive’ and 100 indicated ‘very attractive’.

#### Sexuality variables

Participants’ sexual satisfaction was assessed using the following item: “thinking about your sex life in the last year, how much do you agree or disagree with the following statement: ‘I feel satisfied with my sex life’”. Response options ranged from 1 (strongly agree) to 5 (strongly disagree).

Participants also indicated whether they were attracted to men, women, neither, or both, their current relationship status, and their number of lifetime sexual partners. Four further yes/no questions were asked: “as far as you know, have you ever had an STI?”, “as far as you know, do you currently have an STI?”, “as far as you know, are you allergic or sensitive to latex, non-latex condoms and/or lubricants?” and “have you used a condom the last time you had sex?” Finally, participants were asked: “which one of the following percentages describes better the proportion of occasions of intercourse you have not used a condom in your lifetime?”, “which one of the following percentages describes better the proportion of occasions of intercourse you have not used a condom in the past six months?” and “how easy would it be for you to identify whether a man has an STI, without asking?”. Answers ranged from 0% to 100%, and were grouped into six intervals: 0–10%, 11–30%, 31–50%, 51–70%, 71–90%, and 91–100%.

#### Ratings of facial photographs

Participants were asked to provide five ratings for each of 20 men on the basis of a single photograph of the man’s face taken from the Psychological Image Collection at Stirling (PICS) database [21]: “Please rate the attractiveness of the following man” (*A*); “If you were single, how likely would you be to have sex with this man should the opportunity arise?” (*S*); “If you were single and you were to have sex with this man, how likely is it that you would use a condom?” (*C*); “Out of 100 women like you, how many would have condomless sex with this man should the opportunity arise?” (*M*); and “How likely is this man to have an STI?” (*I*). Participants indicated their answer to each question by moving a slider between 0 and 100. Prior to commencing the task, a simultaneous presentation of all 20 faces was shown to enable participants to anchor their judgements.

#### Stimuli selection and procedures

The Psychological Image Collection at Stirling (PICS) database [21] includes various datasets. The dataset “Aberdeen” was used for this study, which includes 687 colour faces from Ian Craw at Aberdeen. The twenty pictures used for this study were chosen at random to avoid biases.

#### Condom Use Resistance Tactics Scale

The Condom Use Resistance Tactics Scale (T) has obtained strong evidence of reliability and validity [18]. Thirty response items were used in order to answer the question “Since the age of 14, how many times have you successfully avoided using a condom with a man who wanted to use one by”. Example items include “Getting him so sexually excited that he agreed to have sex without a condom” and “Telling him how upset you would be if you did not have sex because you did not have a condom”. The items describe a variety of resistance tactics and can be categorised into ten subscales, each with three items: Reassurance (e.g., ‘‘Reassuring him that you were ‘clean’ so that he would have sex without a condom”, α = .80), Seduction (e.g., ‘‘Getting him so sexually excited that he agreed to have sex without a condom”, α = .89), Sensitivity (e.g., ‘‘Telling him you didn’t want to use a condom because sex doesn’t feel as good with one on”, α = .92), Direct Request (e.g., ‘‘Asking him to not use a condom during sex”, α = .90), Relationship and Trust (e.g., ‘‘Telling him that you trusted each other so that he would have sex without a condom”, α = .82), Emotional Consequences (e.g., ‘‘Telling him how angry you would be if he insisted on using a condom”, α = .64), Deception (e.g., ‘‘Pretending that you had been tested and did not have any STDs”, α = .73), Condom Sabotage (e.g., ‘‘Agreeing to use a condom but intentionally breaking the condom when putting it on”, α = .90), Withholding Sex (e.g., ‘‘Refusing to have sex with him if you had to use a condom”, α = .98), and Physical Threat (e.g., ‘‘Preventing him from getting a condom by staying on top of him”, α not available as two of the three elements were not endorsed by participants).

### Procedure

After providing electronic informed consent, each participant completed the self-administered online questionnaire (taking between 25 and 30 minutes). This protocol was approved by the Ethics Committee of the University of Southampton (Ergo ref: 25115).

### Data analysis

IBM SPSS software (version 25) was used to perform the statistical analysis. To identify factors influencing condom use and interactions amongst them, a series of bivariate associations (Pearson’s correlation coefficients) were calculated, where the assumptions for the test were met (level of measurement, related pairs, absence of outliers, normality of variables, linearity, and homoscedasticity). In analysing participants’ ratings, the data were considered in two ways: first, by considering the data associated with each of the male faces rated, aggregated over the participants’ ratings, and, second, considering the data associated with each participant, aggregated over the men that she rated. For example, we first examined whether some men were judged to be more attractive than others on average, considering the participants as a group. This set of values is denoted by A_M_ (see Table 1 for ratings). Second, we assessed whether some participants found the set of 20 men in the study more attractive than did other participants, considering the men as a group. This set of values is denoted by A_P_.

**Table 1:** The mean participant ratings for each male photograph (Scale 0–100). The standard deviation is shown in parentheses.

Man	Attractiveness (A_M_)	Condom Use Intentions (C_M_)	STI Likelihood (I_M_)	Other Women: Sex Without A Condom (M_M_)	Willingness to have Sex (S_M_)
**1**	25.5 (24.8)	87.9 (26.5)	44.3 (23.0)	18.8 (23.5)	16.3 (23.4)
**2**	15.20 (19.9)	90.3 (24.5)	50.9 (25.2)	14.8 (22,0)	9.0 (18.9)
**3**	19.7 (21.3)	88.8 (25.5)	45.8 (24.3)	16.3 (22.5)	13.0 (19.9)
**4**	24.9 (23.8)	88.0 (25.7)	48.5 (23.1)	19.6 (23.3)	15.3 (21.0)
**5**	27.3 (25.3)	87.9 (24.8)	51.4 (24.0)	19.8 (23.3)	18.5 (24.2)
**6**	19.5 (22.5)	89.3 (25.5)	56.3 (26.4)	16.3 (22.9)	13.4 (21.8)
**7**	22.5 (22.0)	88.4 (25.0)	40.9 (23.0)	17.4 (22.0)	14.8 (21.3)
**8**	15.7 (21.0)	88.9 (26.2)	37.0 (24.3)	13.6 (20.6)	9.3 (18.0)
**9**	12.8 (18.2)	89.4 (26.3)	38.4 (25.5)	12.5 (20.0)	7.9 (16.6)
**10**	38.7 (26.6)	87.8 (23.2)	42.9 (22.2)	25.7 (26.2)	28.4 (28.0)
**11**	13.6 (19.2)	88.2 (27.4)	47.3 (24.4)	13.1 (20.5)	8.5 (17.5)
**12**	17.4 (19.2)	88.1 (26.2)	50.4 (25.0)	15.1 (21.4)	10.0 (18.5)
**13**	14.2 (18.5)	88.8 (26.3)	37.9 (23.9)	13.8 (20.7)	9.5 (18.4)
**14**	12.8 (17.5)	88.4 (27.0)	41.6 (24.6)	12.8 (19.6)	7.9 (16.3)
**15**	28.3 (24.9)	87.2 (25.3)	44.0 (22.6)	20.0 (23.2)	20.8 (25.7)
**16**	26.1 (24.4)	86.9 (26.6)	46.2 (23.6)	19.4 (23.7)	18.9 (24.6)
**17**	27.1 (24.3)	85.3 (28.1)	47.4 (22.9)	20.2 (24.5)	18.2 (23.3)
**18**	14.4 (19.5)	87.8 (27.8)	49.7 (24.2)	13.3 (20.3)	7.4 (15.8)
**19**	24.2 (24.2)	88.8 (24.9)	47.7 (23.3)	17.7 (22.3)	15.4 (22.1)
**20**	17.9 (20.5)	88.9 (26.2)	45.0 (24.3)	15.1 (22.3)	10.4 (17.4)

A generalized linear mixed model (GLMM) with repeated measures was constructed in order to carry out a multivariate analysis addressing the question “What linear combination of factors best explains the variation in participants' condom use intentions across the 20 men rated?” The main benefit of a GLMM is that it enables an examination that incorporates repeated measures (in this case the ratings of the 20 men) and individual variance in participants’ condom use intentions. The GLMM assumptions for homogenous, normal, and independent deviations were evaluated.

## Results

### Demographics

Participants mean age was 19.7 years old *(SD =* 1.4, range = 18–32). In terms of nationality, 353 (74%) participants were Canadian, 38 (8%) were British and the remainder identified as residents from various European (e.g., France), South American (e.g., Colombia), African (e.g., South Africa) and Asian (e.g., China) countries.

### Sexual experience variables

In response to the statement: “I feel satisfied with my sex life”, 102 (21.3%) participants agreed strongly, 194 (40.4%) agreed, 80 (16.7%) neither agreed nor disagreed, 70 (14.6%) disagreed, 21 (4.4%) disagreed strongly and 13 (2.7%) preferred not to say. Four hundred and twenty three (88.1%) participants reported that they were exclusively attracted to men, 50 (10.2%) reported that they were attracted to both men and women and 2 (0.4%) reported that they were not attracted to either men or women. Two hundred and thirty-five (49%) participants were single, 227 (47.3%) were in an exclusive relationship, 7 (1.5%) were in an open relationship, 2 (0.4%) were married and 5 (1%) chose ‘other’. One participant (0.2%) reported currently having an STI, and 20 (4.2%) participants reported having had an STI in the past. The majority of participants (89%) reported at least one sexual partner. Of these, the median number of lifetime sexual partners was 2 (min = 1, max = 30) and the median age at first sexual intercourse was 17 years old (min = 12, max = 23). Twenty-one (4.4%) participants reported an allergy to latex, non-latex condoms and/or lubricants. Reported rates of condomless sexual intercourse are presented in Table 2.

**Table 2:** The percentage of sexual intercourse episodes in which condoms were NOT used reported by participants (excluding participants who did not respond to these questions and those who had not yet had sex) during their lifetime, during the last 6 months and the last time they had sex.

% Condomless Sex	< 10%	< 30%	< 50%	< 70%	< 90%	≤ 100%
Lifetime	201 (41.9%)	52 (10.8%)	35 (7.3%)	57 (11.9%)	70 (14.6%)	58 (12.1%)
Past 6 Months	236 (49.2%)	23 (4.8%)	30 (6.3%)	15 (3.1%)	43 (9%)	120 (25%)
	Condom Not Used			Condom Used		
Last time	226 (47.1%)			235 (49%)		

#### Condom resistance tactics

Half of the women (n = 240) reported using at least one tactic. The four most frequently reported condom resistance tactics were the following: “Getting him really aroused and then starting to have sex without a condom” (*n* = 129), “Getting him so sexually excited that he agreed to have sex without a condom” (*n* = 80), “Reassuring him that you were clean (i.e., did not have any STIs) so that he would have sex without a condom” (*n* = 78) and “Telling him you didn’t want to use a condom because sex doesn’t feel as good with one on” (*n* = 73).

### Participants’ ratings

#### Associations between participants’ ratings of the 20 men

We constructed average ratings for each man and considered relationships amongst these. The more attractive a man was judged to be on average, A_M_, the more likely participants would be willing to have sex with him, S_M_ (*r* = 0.987, df = 478, *p*<0.001). Further, the more attractive a man was judged to be, A_M_, the less likely women were to intend to use a condom during sex, C_M_ (*r* = -0.552, df = 478, *p* = 0.007). Consequently, average condom use intentions, C_M_, tended to be lower for men that participants were, on average, more willing to have sex with, S_M_ (*r* = -0.542, df = 478, *p* = 0.009).

On average, participants judged that more women like themselves would have sex without a condom, M_M_, with the men that they judged, on average, to be more attractive, A_M_ (*r* = 0.993, df = 478, *p*<0.0001), and with whom they were, on average, more willing to have sex (S_M_) (*r* = 0.980, df = 478, *p*<0.0001). Consequently, where the average judgement of the number of women willing to have condomless sex with a man, M_M_, was high, participants’ average condom use intentions towards the man, C_M_, were lower (*r* = -0.541, df = 478, *p* = 0.008).

However, the average perceived STI likelihood for a man, I_M_, had no significant association with average condom use intentions towards him, C_M_, or with his average perceived attractiveness, A_M_, or with participants’ average willingness to have sex with him, S_M_. These bivariate associations are displayed in Table 3.

**Table 3:** Bivariate associations between mean ratings for twenty men (df = 478) of their attractiveness, A_M_, condom use intentions towards them, C_M_, their STI likelihood, I_M_, the extent to which women like the participants would be willing to engage in condomless sex with them, M_M_, and the willingness of the participants to have sex with them, S_M_. Pearson’s *r* values are shown in the upper right half of the table, Spearman’s *ρ* in the lower left: * = p<0.05, ** = p<0.01, *** = p<0.001, grey cells = n.s.

*ρ* / *r*	Attractiveness (A_M_)	Condom Use Intentions (C_M_)	STI Likelihood (I_M_)	Other Women: Sex Without A Condom (M_M_)	Willingness to have Sex (S_M_)
**Attractiveness (**A_M_**)**	-	-0.552**	0.130	0.993***	0.987***
**Condom Use Intentions (**C_M_**)**	-0.648**	-	-0.055	-0.541**	-0.542**
**STI Likelihood (**I_M_**)**	0.189	-0.111	-	0.170	0.085
**Other Women: Sex Without A Condom (**M_M_**)**	0.982***	-0.642**	0.224	-	0.980***
**Willingness to have Sex (**S_M_**)**	0.970***	-0.600**	0.102	0.961***	-

#### Overall ratings of men

For each participant, we averaged their ratings of the 20 men and evaluated relationships amongst these overall ratings. Participants who tended, overall, to rate the twenty men as more attractive, A_P_, also tended to be more willing to have sex S_P_ (*r* = 0.766, df = 478, *p*<0.001) and were less willing to use a condom C_P_ (*r* = -0.150, df = 478, *p*<0.001). Participants who judged that women like themselves would be more willing, overall, to have condomless sex with the twenty men, M_P_, also tended to believe that the twenty men had a higher likelihood of having an STI, I_P_(*r* = 0.156, df = 478, *p*<0.001) and themselves had lower overall condom use intentions, C_P_(*r* = -0.300, df = 478, *p*<0.001), higher willingness to have sex, S_P_ (*r* = 0.643, df = 478, *p*<0.001) and higher judgements of attractiveness overall, A_P_ (*r* = 0.555, df = 478, *p*<0.001). Overall judgement of STI likelihood, I_P_, was also positively correlated with higher overall condom use intentions, C_P_ (*r* = 0.111 df = 478, *p*<0.05).

#### Influence of perceived own attractiveness and ability to detect STIs

The average value for own attractiveness ratings was 67.23 (SD = 20.2). The more attractive a participant judged herself to be, O_P_, the more she believed that, overall, men are likely to have a STI, I_P_(*r* = 0.103, df = 478, *p*<0.05).

Participants’ confidence in their ability to detect whether a potential sexual partner had an STI without asking was significantly negatively correlated with their overall willingness to have sex, S_P_ (*r* = 0.163, df = 478, *p*<0.001), positively correlated with the likelihood of having an STI, I_P_(*r* = 0.122, df = 478, *p* = 0.008), and was also associated with overall lower condom use intentions in themselves, C_P_ (*r* = -0.227, df = 478, *p*<0.001), and women like themselves, M_P_ (*r* = 0.125, df = 478, *p* = 0.006).

#### Influence of age, nationality and sexual experience variables

Age did not correlate with attractiveness ratings, willingness to have sex, condom use intentions or STI likelihood. Nationality did not correlate with attractiveness ratings, willingness to have sex or condom use intentions, but it presents significant results with STI likelihood and condom use intentions of other women (see Table 4). Sexual experience variables (such as reported condom use) showed significant trends. These relationships are displayed in Table 4.

**Table 4:** Bivariate associations (Pearson’s *r*) between 480 (df = 478) participant demographic and sex experience variables (left column) and their mean ratings of 20 men. Significance levels are indicated: * = p<0.05, ** = p<0.01, grey cells = n.s.

*R*	Attractiveness (A_P_)	Condom Use Intentions (C_P_)	STI Likelihood (I_P_)	Other Women: Sex Without A Condom (M_P_)	Willingness to have Sex (S_P_)
**Age**	.001	.047	-.086	-.066	-.034
**Nationality**	.004	-.049	-.120**	.105*	.008
**Satisfaction with Sex Life**	-.126**	-.005	.034	-.050	-.126**
**No. Sex Partners**	.014	-.036	-.015	.082	.040
**Relationship Status**	-.064	-.113*	-.011	-.078	-.054
**Past STI**	-.028	.041	.036	.015	-.012
**Present STI**	-.042	.030	.011	-.021	-.035
**Age at First Intercourse**	.017	.117*	-.100*	-.063	-.038
**Condomless Sex In Lifetime**	.011	-.142**	.086	.134**	.054
**Condomless Sex In Last 6 Months**	-.064	-.126**	.064	.074	-.016
**Condomless sex with more than 2 partners in the past 6 months**	.001	-.148**	.097*	.115**	.090
**Condom use last time you had sex**	.074	.061	-.096*	-.024	.020
**Latex allergy**	-.058	-.014	-.014	.015	-.059

#### Influence of condom resistance tactics

Of the 30 items considered, some showed significant correlations with attractiveness, sex and condom use intentions, sexual health status and own perceived attractiveness; the majority, however, did not show any strong associations (see Table 5).

**Table 5:** Bivariate associations (Pearson’s *r*) between 480 (df = 478) condom resistance tactics factors (left column) and their mean ratings of 20 women. Significance levels are indicated: * = p<0.05, ** = p<0.01, grey cells = n.s.

*R*	Condom Use Intentions (C_P_)	Attractiveness (A_P_)	Willingness to have Sex (S_P_)	Other Women: Sex Without A Condom M_P_)	STI Likelihood (I_P_)	Own attractiveness (O_P_)
**Seduction**	-.125**	.017	.063	.086	.094*	.181**
**Reassurance**	-.027	-.014	.045	.040	.085	.090*
**Sensitivity**	-.011	.004	.003	.040	.039	.068
**Direct Request**	-.041	.013	.068	.062	.018	.049
**Relationship Trust**	-.078	-.030	.000	.054	.100*	.085
**Emotional Consequences**	.017	.019	.041	.050	.071	.068
**Deception**	-.052	.035	.067	.056	.030	.019
**Sabotage**	-.085	.113*	.136**	.100*	.058	-.013
**Withholding sex**	-.096*	.126**	.134**	.108*	.067	-.009
**Physical Threat**	-.113*	.084	.120**	.072	-.011	.105*

### Linear mixed model

Participant condom use intention ratings was the outcome variable, with the repeated measures being the individual men rated. All demographic and sexual experience variables and rating variables were included as main effects. The model thus attempted to identify a single set of relationships that could account for all participants' patterns of condom use intentions.

Women showed significantly higher condom use intentions with: men who they rated as less attractive (p<0.0005), men who they rated as less likely to carry or transmit an STI (p<0.0005), men with whom they were less interested in having sex (p<0.0005) and when they estimated that fewer of their peers would also have condomless sex with him (p<0.0005). Demographic and sexual experience variables did not emerge as significant predictors, except for the percentage of condomless sex percentage in their lifetime (*p* < .05).

## Discussion

The results of the current study demonstrated a strong association between perceived attractiveness (of a potential partner and of self) and condom use intentions in women who have sex with men. Participants were more willing to have sex with more attractive men, but were less inclined to use condoms when they do so. These findings agree with those of a previous study [5] on the influence of attractiveness on condom use intentions in a heterosexual male population. The findings are also in agreement with previous work that has highlighted that individuals use unimportant or irrelevant factors to judge partners’ relative safety [22] [23] and that different contextual factors, like relationship motivation and partner familiarity can be used to justify sexual risk taking [24].

Studies have demonstrated that people form beliefs about STI risk during first encounters [25], that these judgements can be made within milliseconds [26], and that they are based on a wide variety of factors [27]. However, prior to this study, the influence of women’s confidence in their judgements on STI risk and condom use intentions had not been systematically investigated. We found no overall relationship between judgements of STI likelihood and judgements of partner attractiveness, as was also the case for heterosexual men in a previous study by Eleftheriou et al. [5]. This result was not consistent with the study by Rupp et al. [10], which suggested that women have a sexual preference for high-risk men. In the current study, participants’ confidence in their own ability to judge whether a potential sexual partner is infected with an STI on the basis of appearance was significantly positively correlated with their tendency to be willing to have sex and with overall lower condom use intentions than participants with lower confidence. However, this result was not confirmed by the GLMM.

Moreover, in the current study, we found that participants reported lower condom use intentions towards men with whom they were willing to have sex. This result was surprising when we considered that these same women also judged that a greater number of women like themselves would also be willing to have condomless sex with these men. This judgement should imply that these men were at higher risk for STI transmission, since they would presumably be engaging in more condomless sex with more partners (other women like the participant). However, this observation did not translate into higher perceived risk in terms of increased overall condom use intentions towards more attractive men, or a correlation between attractiveness and STI likelihood. This finding may be more easily explained, when we consider the work of Fishbein et al. [7] and Williams et al. [28], who found that risk information about a partner is sometimes ignored when the partner is attractive. Moreover, this finding also seemed more logical when we considered that each participant perceived themselves as unlikely to currently be infected with an STI (in fact, only 4% of our sample had ever been diagnosed with an STI). Thus, when a participant imagined 100 women like herself, it is likely that she similarly estimated that these women would also be unlikely to be an STI transmission risk [29]. Though this was beyond the scope of the current work, this may help explain a possible reason why participants failed to perceive this risk cue.

By improving our understand of the link between condom use and perceived attractiveness we anticipate that the current findings will contribute to improvements in the subsequent design of a sexual education interventions. It is essential to help individuals to recognize their misconceptions and reflect on their intentions compared to their actual behavior. Individuals need to be well informed about the many different ways in which their judgements and decisions regarding sexual risk taking can be influenced and impaired. It may be useful to explore interventions [30] that target the tensions between some of the beliefs exhibited by the participants in the current study. The fact that individuals often underestimate their probability of facing unpleasant events or outcomes could be interpreted in terms of unrealistic optimism [31] and could be addressed appropriately using a sex education intervention. For example, a virtual reality game that focuses on the users and their immersion and engagement with a simulated population could potentially challenge the users’ perception of invulnerability, as they encounter various people and scenarios that affect their health throughout the game [32].

Future research could investigate whether individual differences in variables known to influence risk taking, such as sexual sensation seeking [33] and sexual excitation/inhibition [34], might mediate the relationship between attractiveness and condom use intentions.

### Strengths and limitations

The degree to which participants were sexually aroused was not recorded during the study. Because sexual arousal can negatively influence condom use intentions in women [24], this aspect may play a role in how women respond to attractive male partners. Moreover, the fact that some women might have been using hormonal contraception, which might affect condom use intentions [35], was not investigated. A consistent finding in the literature is that when people are in committed relationships, there is often a shift from condom use to hormonal contraception [36]. Women who were not exclusively attracted to men, were also included in the current analyses; unfortunately we did not obtain a sufficient sample size to compare these women with women who reported an exclusive attraction to men. Thus, we are not able to speak to any group differences based on sexual orientation in the current work. Future research should include greater diversity in their samples. A single-item measure was used to rate sexual satisfaction, instead of a validated scale. Another limitation was the relatively homogeneous sample and the fact that this was primarily a student sample and their knowledge and attitudes may not generalise to other populations (there is a risk of possible selection bias on age, background and nationality). However, evidence from previous studies suggests that student samples do not intrinsically pose a problem for a study’s external validity [37] and also STIs and HIV pose a considerable and increasing health threat among young people [38]. Finally, participants’ reported condom use intentions in this study may or may not resemble their actual usual condom use behaviour [39] due to the influence of contextual factors such as alcohol. On the other hand, previous research has shown evidence that condom use was related to intentions [40] and therefore, intentions are worth investigating.

Notwithstanding these limitations, this study is the first to explore the relationship between perceived attractiveness and condom use intentions in women who have sex with men, including their self-ratings of attractiveness, previous sexual experiences, and condom resistance tactics.

## Conclusions

In summary, this is the first study that investigated the association between own perceived attractiveness, sexual health status, condom resistance, sex and condom use intentions in a female population. Female perceptions of attractiveness influence their condom use intentions; such risk biases could profitably be considered and discussed during sex and relationships education sessions in educational settings.

## Supporting information

S1 Dataset (XLSX)
